# Supplementary material for: Intrinsic room-temperature ferromagnetism in a two-dimensional semiconducting metal-organic framework
Source: Nat Commun. 2023 Nov 3;14:7063. doi: 10.1038/s41467-023-42844-9 (PMC10624846; doi:10.1038/s41467-023-42844-9)
Supplement: Supplementary file 1 — Supplementary Information [file 41467_2023_42844_MOESM1_ESM.pdf]

Supplementary Information

Intrinsic room-temperature ferromagnetism in a two-dimensional semiconducting metal-organic framework

Sihua Feng,[1] Hengli Duan,[1,*] Hao Tan,[1] Fengchun Hu,[1] Chaocheng Liu,[1] Yao Wang,[1] Zhi Li,[1] Liang Cai,[1] Yuyang Cao,[1] Chao Wang,[1,*] Zeming Qi,[1] Li Song,[1] Xuguang Liu,[2] Zhihu Sun[1] and Wensheng Yan[1,*]

[1]National Synchrotron Radiation Laboratory, University of Science and Technology of China, 230026 Hefei, Anhui, China
[2]Hefei National Laboratory for Physical Sciences at the Microscale, University of Sci ence and Technology of China, 230026 Hefei, Anhui, China

Corresponding Author
* Hengli Duan — Email: hlduan@ustc.edu.cn
* Chao Wang — Email: chaowng@ustc.edu.cn
* Wensheng Yan — Email: ywsh2000@ustc.edu.cn

Supplementary Notes

Supplementary Note 1. Structure determination. The presence of the distinct sequences of functionalities along the MOF backbone will cause an abundant pore environment, and not change the crystal structure of the MOFs. Therefore, we synthesized the Cu-ABDC using 2-amino-1,4-benzenedicarboxyulic acid (ABDC) and $Cu(NO_3)_2 \cdot 3H_2O$, and the crystal structure is exactly consistent with that of well-known Cu(tpa)·DMF, which is synthesized using 1,4-benzenedicarboxyulic acid (BDC) and $Cu(NO_3)_2 \cdot 3H_2O$. Then, we obtained the crystal structure data from the Cambridge Crystallographic Data Centre (CCDC-687690), which has been reported by Carson et al.. The crystal structure of the Cu(tpa)·DMF is monoclinic with space group *C*2/*m* (NO. 12). The lattice parameters are a = 11.4143 Å, b = 14.2687 Å, c = 7.7800 Å, β= 108.119°, V = 1204.27 Å^3, respectively. The diffraction patterns of our Cu-ABDC MOF match well with that of Cu(tpa)·DMF MOF, indicating the same crystal structure. Besides, for structure characterizations of the Cu-MOFs, the spin-polarized DFT calculations implemented in the Quantum Espresso software package were performed.

Supplementary Note 2. Photoconductivity measurements. To further verify the semiconducting property, the photoconductivity measurements were also performed. The pressed pellets were prepared by adding about 14 mg samples (heated at 100°C under argon overnight) in a 6 mm inner diameter split sleeve under the applied pressure of 20 MPa. The thickness of the pressed pellet was about 0.37 mm. And Au particles were deposited on the surface of the pressed pellet samples as electrodes via the sputtering method. The photo-electrical properties of the as-fabricated Cu-ABDC were characterized by a probe station using a 4200A-SCS source meter (Keithley) with Clarius software using 2-probe voltage linear scan mode. The incident light source was LED with wavelength of 1000 nm, and the light intensity was calibrated using an optical power meter (Newport Model No. 2936 R). The photoconductivity characterization was carried out in ambient condition by applying a sweeping bias of 10 V at room temperature.

Supplementary Note 3. Electrochemical measurements. Mott-Schottky and solid-state cyclic voltammetry measurements were performed on a CHI760D electrochemical workstation using a typical three-electrode system. The Cu-ABDC nanosheets on glassy carbon electrodes acted as the working electrode with a platinum mesh as the counter electrode and an Ag/AgCl reference electrode. Typically, 4 mg of samples and 30 μL Nafion solution (5 wt%, Sigma Aldrich) were dispersed in 1 mL ethanol solution to form a homogeneous ink assisted by ultrasonic method. Mott-Schottky

measurements were conducted in 1 M KOH (aq) electrolytes continuously purged with 99.999% N_2 (Praxair) and at a sweep rate of 5 mV/s. Then, Mott-Schottky analysis was carried out in the linear region of the C^{-2} curve from 0.9 to 1.3 V vs Ag/AgCl with a frequency of 2 kHz. The solid-state cyclic voltammetry was measured at 0.1 V/s scan rates in 0.1 M $TBAPF_6$/DMF and KCl solutions.

Supporting Figures

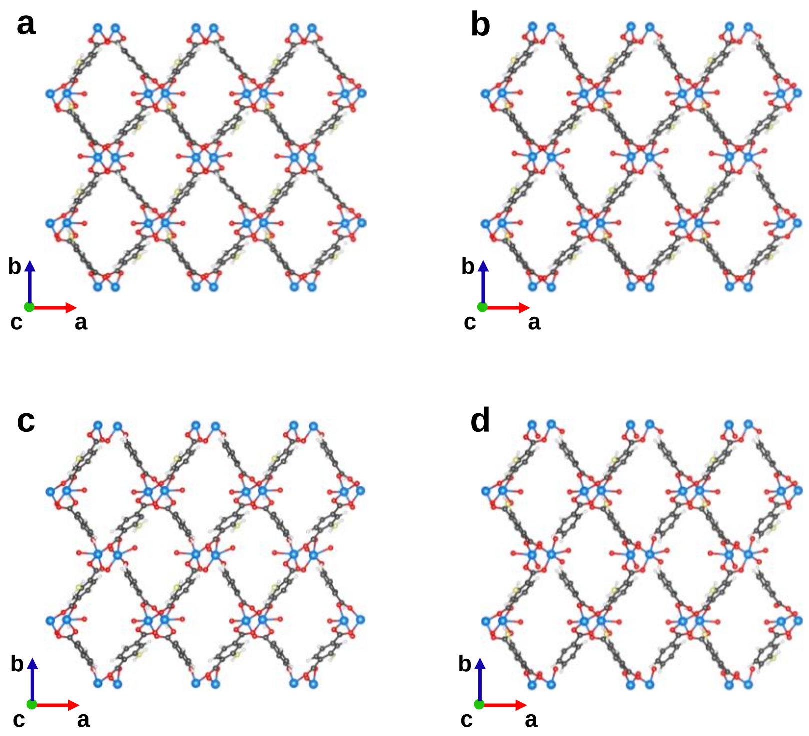

Supplementary Figure 1. Structure models of Cu-MOFs. Structure models of the Cu-ABDC (**a**), 1.4%-LS-Cu-ABDC (**b**) and 2.7%-LS-Cu-ABDC at diagonal (**d**) and adjacent (**d**), respectively. Copper, carbon, nitrogen, oxygen and hydrogen atoms are shown in blue, gray, yellow, red and white, respectively, and the axial solvent molecules are omitted for clarity in all structure models.

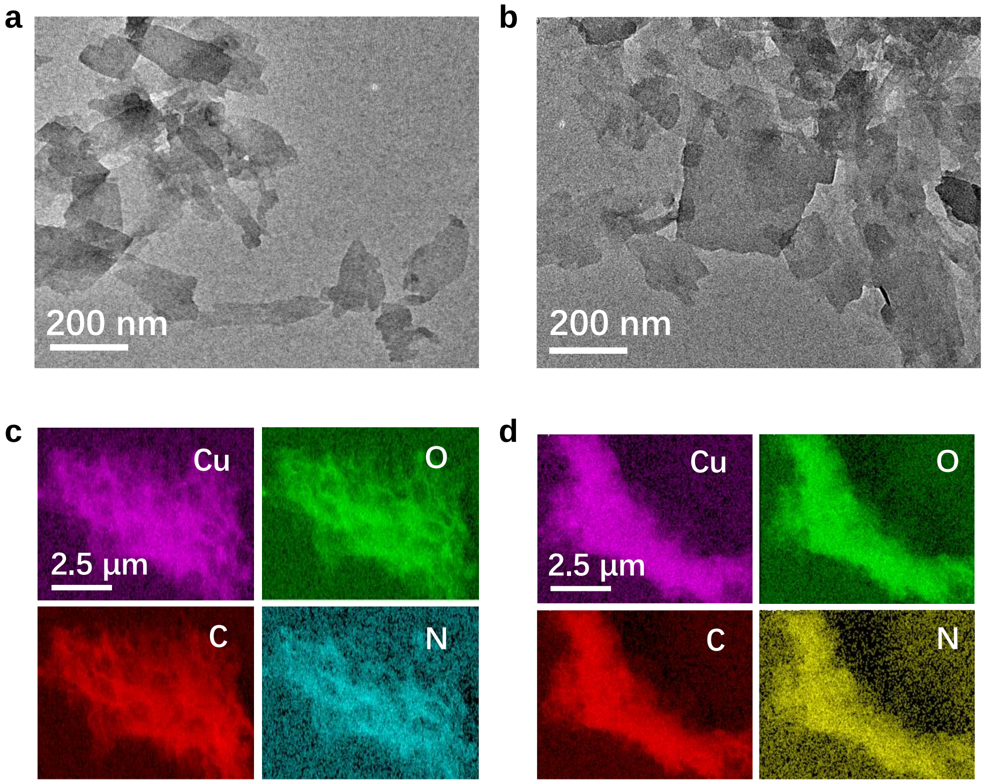

Supplementary Figure 2. TEM characterizations. a-b, TEM images of 1.4%-LS-Cu-ABDC and 2.7%-LS-Cu-ABDC nanosheets. **c-d**, Corresponding mapping images of 1.4%-LS-Cu-ABDC and 2.7%-LS-Cu-ABDC.

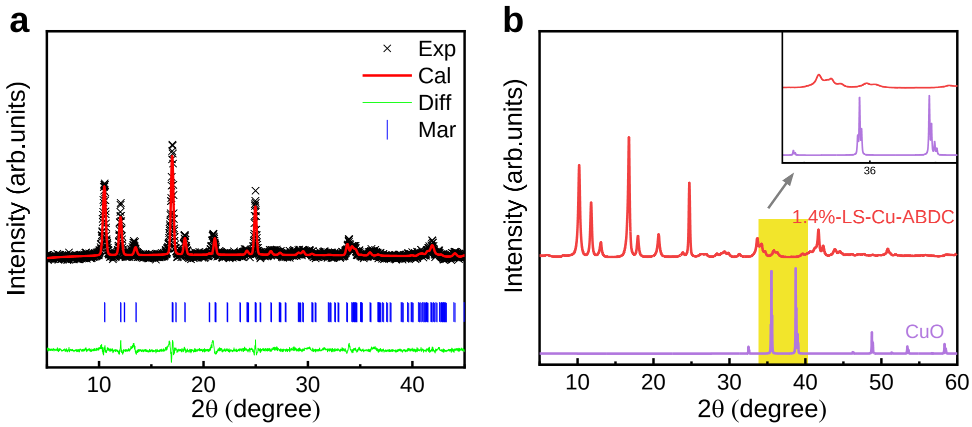

Supplementary Figure 3. XRD characterizations. Rietveld refined XRD pattern of Cu-ABDC (**a**) and synchrotron radiation powder X-ray diffraction patterns of 1.4%-LS-Cu-ABDC and the reference sample CuO (**b**). The black cross, red and green horizontal lines, and blue vertical lines are defined as experimental, calculated, difference data and marked position, respectively. The enlargement was marked as yellow shading.

Considering that the presence of the distinct sequences of functionalities along the MOF backbone will cause an abundant pore environment, and will not change the crystal structure of the MOF.[1-2] Therefore, we synthesized the Cu-ABDC using 2-amino-1,4-benzenedicarboxyulic acid (ABDC) and $Cu(NO_3)_2{\cdot}3H_2O$, and the crystal structure is exactly consistent with that of well-known monoclinic Cu(tpa)·DMF.[3] We also have carried out the Rietveld refined XRD pattern of the Cu-ABDC MOF using the software Rietica. As seen from Figure S3, all peaks of the MOF are indexed to the standard structure Cu(tpa)·DMF MOF with space group *C* 2/m (CCDC-687690), which is synthesized using 1,4-benzenedicarboxyulic acid (BDC) and $Cu(NO_3)_2{\cdot}3H_2O$ and has been reported by Carson *et al.*,[1-5] suggesting that the Rietveld refinement of the XRD data reveals an almost single-phase nature of the MOF. The lattice parameters of the MOF obtained from the refinement are a = 11.1345 Å, b = 14.2439 Å and c = 7.8529 Å, which is basically in agreement with the value reported by Carson *et al.*, indicating a reliable quality for our sample. Additionally, in order to exclude the Cu-related phase in our samples, synchrotron radiation powder X-ray diffraction patterns of 1.4%-LS-Cu-ABDC and the reference sample CuO were also performed. Compared to the characteristic peak at about 35.5 ° and 38.7° for CuO, there are no obvious peaks at the same position for 1.4%-Cu-ABDC sample (Figure S3b), confirming the absence of CuO and hence intrinsic ferromagnetic in our samples.

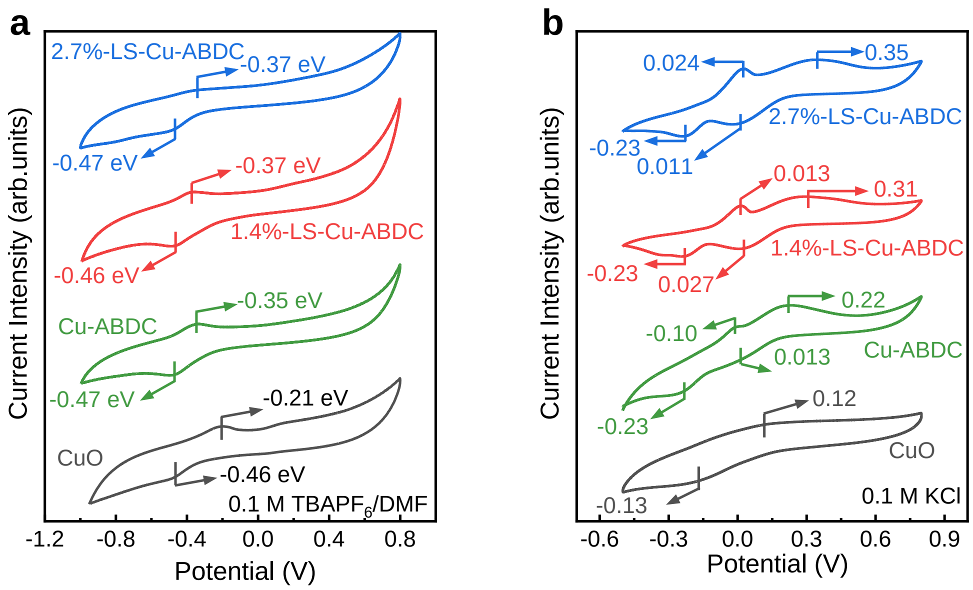

Supplementary Figure 4. Solid-state cyclic voltammetry (CV) measurement. Solid-state cyclic voltammetry (CV) in 0.1 M $TBAPF_6$/DMF (**a**) and KCl solutions (**b**).

The CV curves were measured at 0.1 V/s scan rate in 0.1 M $TBAPF_6$/DMF ($TBAPF_6$, Tetrabutylammonium Hexafluorophosphate) and KCl solutions.[6-7] As shown in Figure S4a, the peaks at about −0.37 and −0.47 V for Cu-MOFs, and at about −0.21 and −0.47 V for CuO were observed in 0.1 M $TBAPF_6$/DMF solution, indicating the successfully synthesized of Cu-MOFs and the absence of CuO. In 0.1 M KCl solution (Figure S4b), two pairs of redox peaks were observed for Cu-ABDC, and only one pair of redox peaks at about 0.12 and −0.13 V for CuO. These different peak positions between CuO and Cu-MOFs indicate the absence of CuO particles.

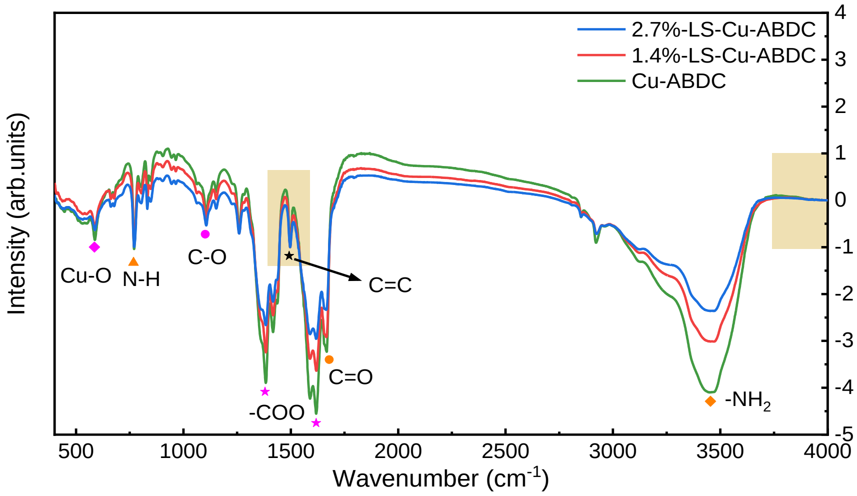

Supplementary Figure 5. FT-IR analysis. The curves of all the samples are normalized by the vibration intensity of C=C (marked as yellow shadings) for the same amount of the organic ligand. For all the Cu-MOFs, the peak at about 3482 cm^{-1} is clearly observed which is ascribed to the asymmetrical stretching vibration adsorption of the amine groups. In the lower frequency region, the peaks at about 1590, 1507 and 1259 cm^{-1} correspond to the N-H, C-H and C-N stretching bonds, respectively. The peaks at about 1101, 1500 and 1668 cm^{-1} are ascribed to the vibrations of C-O, C=C stretching of the benzene ring and C=O of solvent molecule.[8] In addition, two peaks at ~1384 and 1619 cm^{-1} are assigned to the symmetric and asymmetric vibrations of the -COO groups, confirming the existence of ligand in Cu-MOFs skeleton.[9-10] Besides, a characteristic peak at about 580 cm^{-1} can be assigned to Cu-O stretching, suggesting that organic ligands are efficiently coordinated to Cu atoms to form Cu-MOFs. Moreover, the vibration intensities of -COO gradually decrease with the increase of ligand cleavages after the normalization with the signals of C=C, suggesting the missing of organic linkers. Obviously, the vibration intensities of -NH_2 for 1.4%- and 2.7%-LS-Cu-ABDC gradually decrease with the increase of ligand cleavages by about 25% and 42%, respectively, which is close to the actual ratio of benzoic acid. Therefore, the chemical formula for 1,4%-LS-Cu-ABDC and 2.7%-LS-Cu-ABDC samples can be estimated to be $Cu(ABDC)_{0.75}\cdot(BA)_{0.25}\cdot DMF\cdot OH_{0.25}$ and $Cu(ABDC)_{0.58}\cdot(BA)_{0.42}\cdot DMF\cdot OH_{0.42}$, respectively.

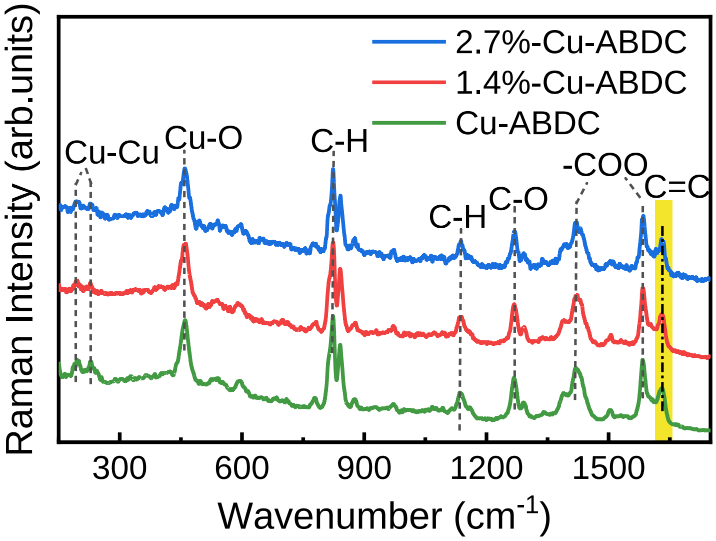

Supplementary Figure 6. Raman spectroscopy analysis. The vibration intensity of all the peaks are normalized by the vibration intensity of C=C (marked as yellow shadings) at about 1633 cm^{-1} for the same amount of the organic ligand. In the low-frequency region, a doublet at about 194 and 229 cm^{-1} are assigned to Cu-Cu stretching modes. At about 460 cm^{-1}, Cu-O stretching mode is detected, which indicates that Cu ions coordinate with ligands and the successfully synthesis of the Cu-MOFs.[11] The peaks at ~820 and 1130 cm^{-1} represent the vibrations of C-H in Cu-MOFs. In addition, a peak at about 1267 cm^{-1} is attributed to C-O bond. Two peaks at about 1420 and 1584 cm^{-1} are dominated by the vibrations of -COO groups, and the band at about 1633 cm^{-1} is assigned to C=C stretching mode.[12-13] Meanwhile, a new band can be discovered at about 1137 cm^{-1}, likely owing to a deformation mode involving the carboxylate group coupled with a C-C stretching mode.[14] It is worth noting that the vibration intensities of -COO gradually decrease with the ligand cleavages, which further confirms the missing organic linkers.

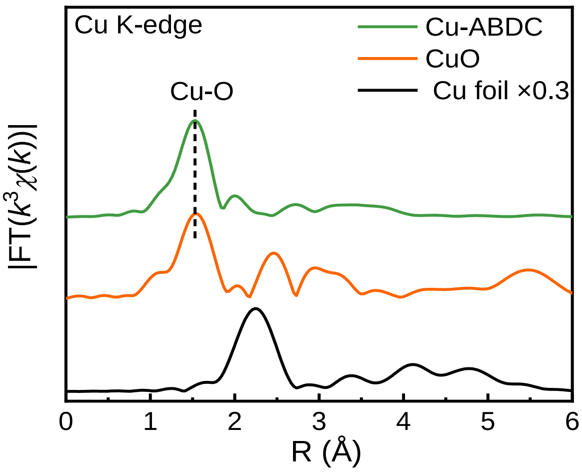

Supplementary Figure 7. Cu K-edge EXAFS in *R*-space. Cu K-edge EXAFS curves including Cu foil and CuO as reference samples indicate the Cu-O coordination in Cu-ABDC.

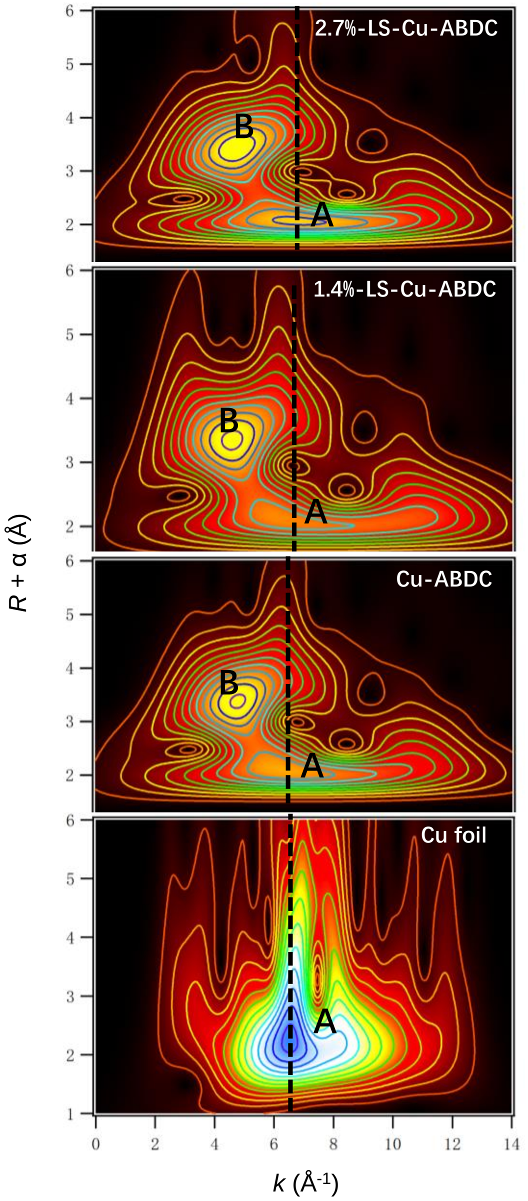

Supplementary Figure 8. Wavelet transform (WT) analysis of Cu-MOFs. After filtering out the coordination of Cu-O in the first shell, the WT of Cu K-edge EXAFS data of Cu-MOFs show a maximum A at the cross-point of about R_A = 2.1 Å/k_A = 6.3 Å^{-1}, which is close to that of Cu foil, confirming that the second shell arises from the coordination of Cu-Cu, in agreement with the EXAFS fitting results. Furthermore, the maximum B at about R_A = 3.4 Å/k_A = 4.3 Å^{-1} may be from the multiple shell scattering or higher shell contribution of Cu-C coordination.

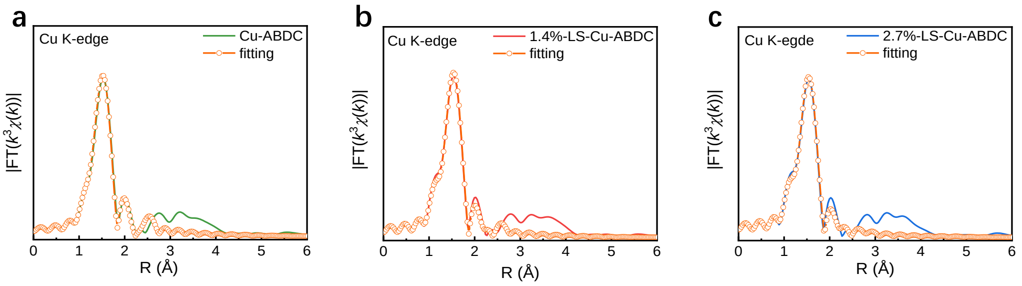

Supplementary Figure 9. Cu K-edge EXAFS fitting curves in *R*-space. Cu K-edge EXAFS fitting curves in *R*-space of Cu-ABDC (**a**), 1.4%-LS-Cu-ABDC (**b**), 2.7%-LS-Cu-ABDC (**c**), respectively. The fitted *R* range here is from 1.1 to 2.4 Angstroms.

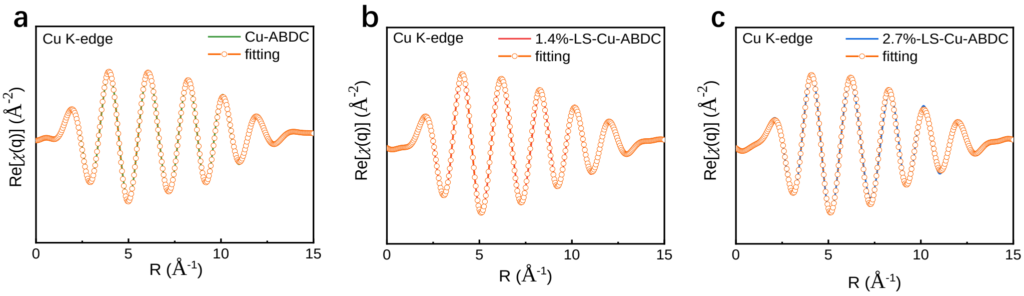

Supplementary Figure 10. Cu K-edge EXAFS fitting curves in *q*-space. Cu K-edge EXAFS fitting curves in *q*-space of Cu-ABDC (**a**), 1.4%-LS-Cu-ABDC (**b**) and 2.7%-LS-Cu-ABDC (**c**), respectively.

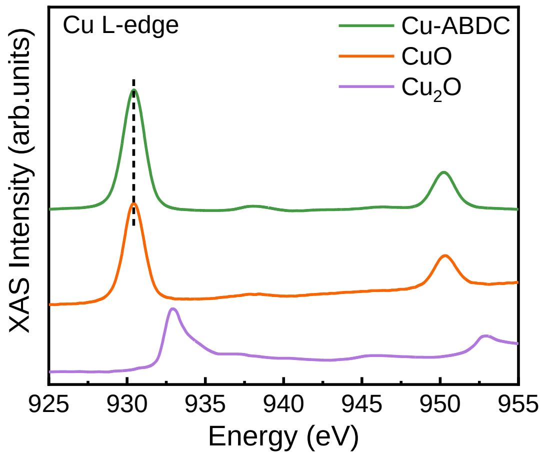

Supplementary Figure 11. Cu L-edge XAS. Cu L-edge XAS for Cu-ABDC including Cu_2O and CuO as reference samples suggests +2 valence state of Cu ions in Cu-ABDC. For Cu L-edge XAS in Fig. 2c, firstly the linear pre-edge background is subtracted, and the L_3 and L_2 edges were normalized on intensities of the corresponding L_2 peaks in order to reveal the variation of the *d*-orbital occupation state more clearly.

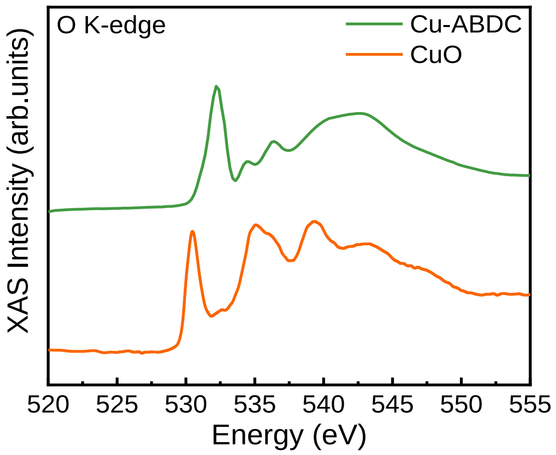

Supplementary Figure 12. O K-edge XAS. O K-edge XAS of Cu-ABDC and the reference sample CuO. Two regions can be directly detected from O K-edge, a sharp π^* region around 530 eV origins from the electrons excited into O 2*p* orbitals hybridized with Cu 3*d*, and the σ* region at about 540 eV is mainly from the electrons excited into O 2*p* orbitals that are hybridized with Cu 4sp and C 2*sp*.[15-17] O K-edge of Cu-ABDC is different from that of CuO because oxygen atoms connect not only with Cu ions but also with benzene ring, which further confirms the synthesis of Cu-ABDC and the absence of CuO. For O K-edge XAS in Fig. 2d, the linear pre-edge background was normalized to greatly exhibit the hybridization between O 2*p* and Cu 3*d* orbitals.

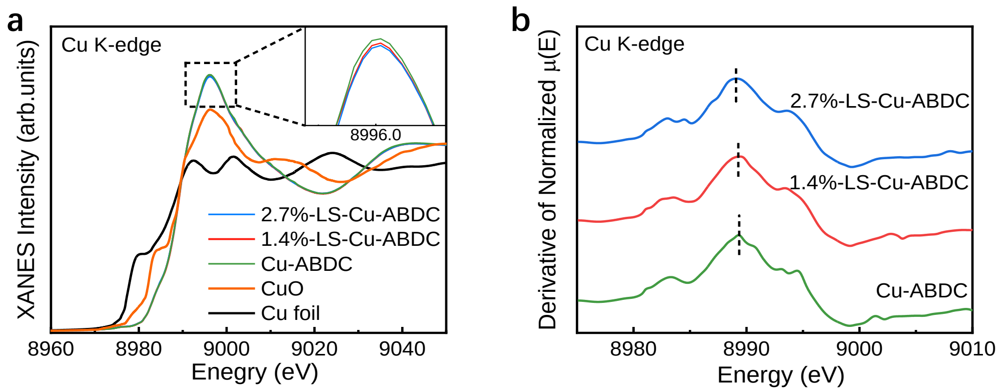

Supplementary Figure 13. Cu K-edge XANES curves. a, Cu K-edge XANES curves of Cu-MOFs and the reference samples. **b**, Cu K-edge first-derivative XANES curves of Cu-MOFs. The absorption edge positions of Cu-MOFs are close to that of CuO, indicating +2 valence state of Cu ions in Cu-MOFs. The decreased absorption intensities of white-line peaks at about 8996 eV and the lower-energy shifts of Cu K-edge first-derivative XANES further confirm that the electron occupation of Cu 3*d* states gradually increase with the increase of ligand cleavage.

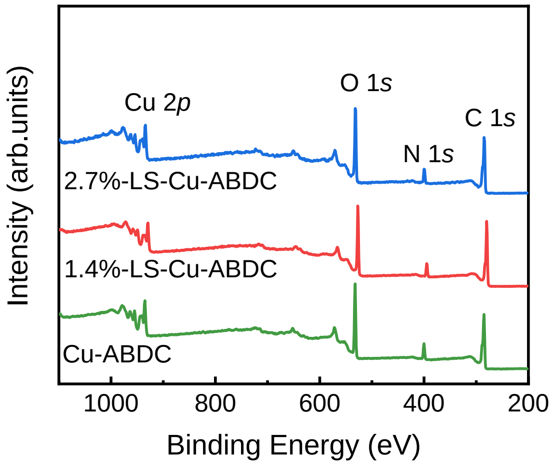

Supplementary Figure 14. **XPS survey analysis**. XPS survey analysis reveals that there are only Cu, N, O and C elements in Cu-MOFs without other magnetic impurities.

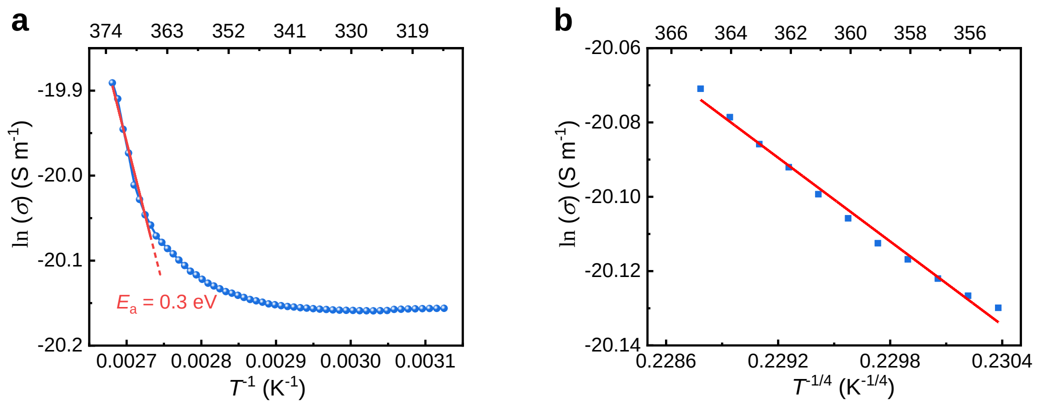

Supplementary Figure 15. The plot of ln σ versus the reciprocal of the temperature (1/T) over the range from 320-373 K (**a**). The plot of ln (σ) versus $T^{-1/4}$ over the temperature region 355-365 K (**b**). The dotted line is the experiment data, and the solid line is the fitting curve.

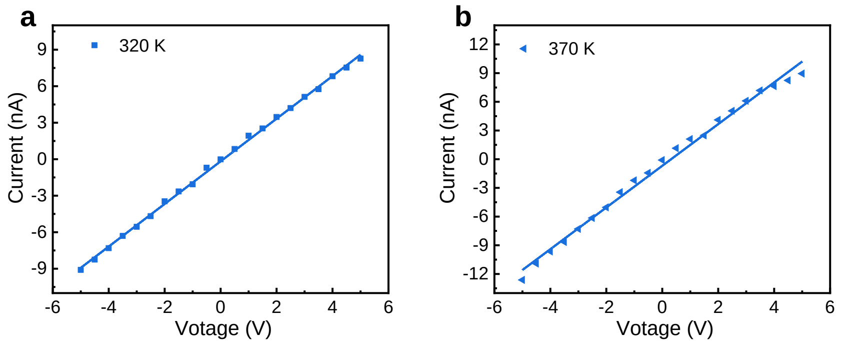

Supplementary Figure 16. *I*-*V* curves for Cu-ABDC collected at 320 K and 370 K.

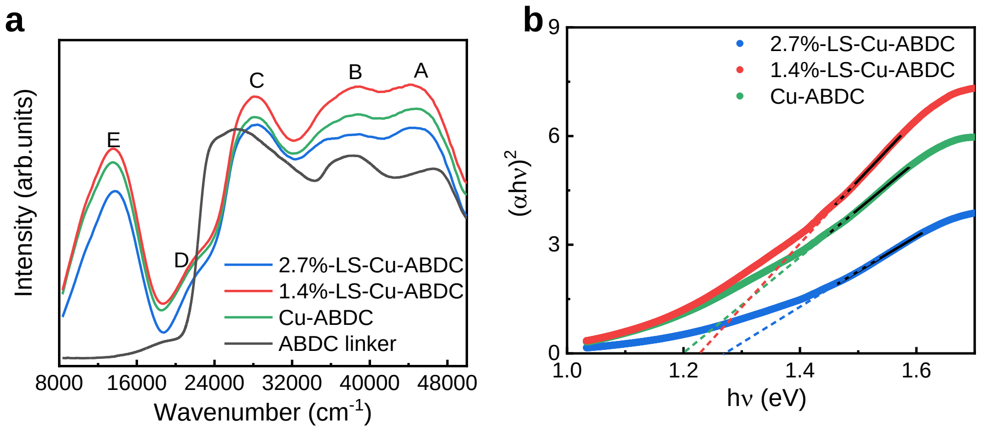

Supplementary Figure 17. UV-Vis-NIR spectra of Cu-MOFs. UV-Vis-NIR spectra of Cu-MOFs and ABDC linker (**a**). Tauc plot derived from absorption of Cu-MOFs (**b**).

Two absorption peaks D (21453 cm^{-1}) and E (13609 cm^{-1}) can be detected, which are attributed to charge transfer between ligand and metal and metal-radical spin-exchange originated from *d-d* transition.[18] There also present three peaks A (45610 cm^{-1}), B (38021 cm^{-1}) and C (27934 cm^{-1}), which may be ascribed to the transition of the $\pi\rightarrow\pi^*$ of the organic functional groups, the transition of the $\pi\rightarrow\pi^*$ of the aromatic rings and the electron transfer transition from ligand to metal.[14, 19-20] Moreover, bandgaps are fitted as 1.20, 1.22 and 1.27 eV for Cu-ABDC, 1.4%-LS-Cu-ABDC and 2.7%-LS-Cu-ABDC, respectively, suggesting its semiconductor behaviors and the bandgaps gradually increase with the ligand cleavages, in agreement with theoretical calculation results below. Remarkably, the UV-Vis-NIR spectra for all the Cu-ABDC samples exhibit absorption at about 13609 cm^{-1} extending to the near-infrared (NIR) region. However, this absorption band was not found in the ABDC linker, indicating a strong *d-π* conjugation between the metal node and the organic linkers, which means the existence of exchange interaction between the metal nodes and the organic linkers.[21-22]

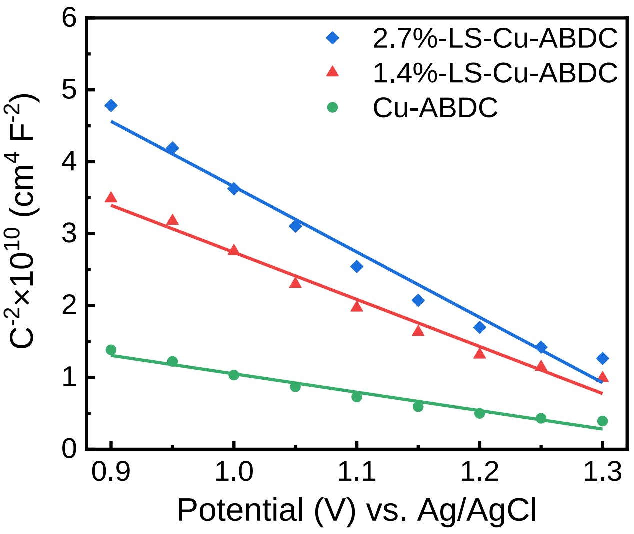

Supplementary Figure 18. Mott-Schottky plots of Cu-MOFs. Mott-Schottky analysis was carried out in the linear region of the C^{-2} curve from 0.9 to 1.3 V vs Ag/AgCl with a frequency of 2 kHz in 1 M KOH.[23-24] Typical negative slopes of the linear region in the Mott-Schottky plots can be founded, indicating all the Cu-MOFs exhibit *p*-type semiconductor character,[25] in agreement with theoretical calculation results. Additionally, the carrier concentration is inversely proportional to the slope of the plots and can be calculated from the function as stated in previous report.[23] Therefore, the hole carrier density of Cu-ABDC is about 2.5 and 3.5 times larger than that of the 1.4%-LS-Cu-ABDC and 2.7%-LS-Cu-ABDC, respectively.

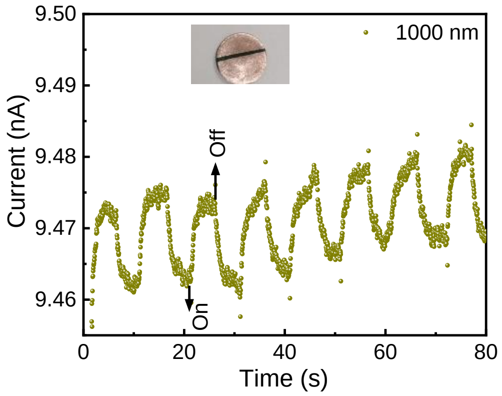

Supplementary Figure 19. **Photoconductivity measurements.** The photo-response of Cu-ABDC under 1000 nm illumination. The photoconductivity characterization was carried out in ambient condition by applying a sweeping bias of 10 V at room temperature. As shown in Figure S19, the current values increase under 1000 nm illuminations, further indicating the semiconducting character of Cu-ABDC.[26-29]

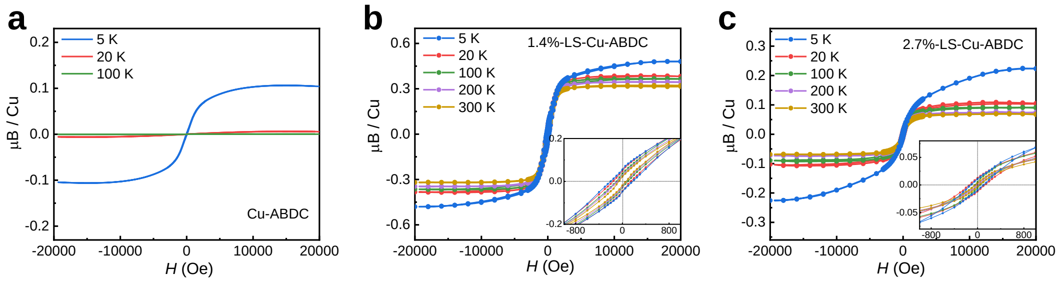

Supplementary Figure 20. The *M-H* curves at different temperatures for Cu-MOFs. *M-H* curves of Cu-ABDC (**a**), 1.4%-LS-Cu-ABDC (**b**) and 2.7%-LS-Cu-ABDC (**c**), respectively.

Distinct hysteresis loops were observed for 1.4%- and 2.7%-LS-Cu-ABDC MOFs in the temperature range from 5 to 300 K, as shown in Figure S20b-c. The remanent magnetization (M_r) is about 0.06 μ_B/Cu with the coercive field of about 170 Oe at 5 K, and 0.02 μ_B/Cu with a coercive field of 60 Oe at 300 K for 1.4%-Cu-ABDC MOF. For 2.7%-Cu-ABDC MOF, the M_r is about 0.01 μ_B/Cu and 0.003 μ_B/Cu at 5 K and 300 K, with a coercive field of about 100 Oe and 35 Oe, respectively. The relatively high remanent magnetization (about 12% at 5 K for 1.4%-Cu-ABDC MOF) at finite temperature indicates the presence of spontaneous magnetization, and hence the ferromagnetic order.

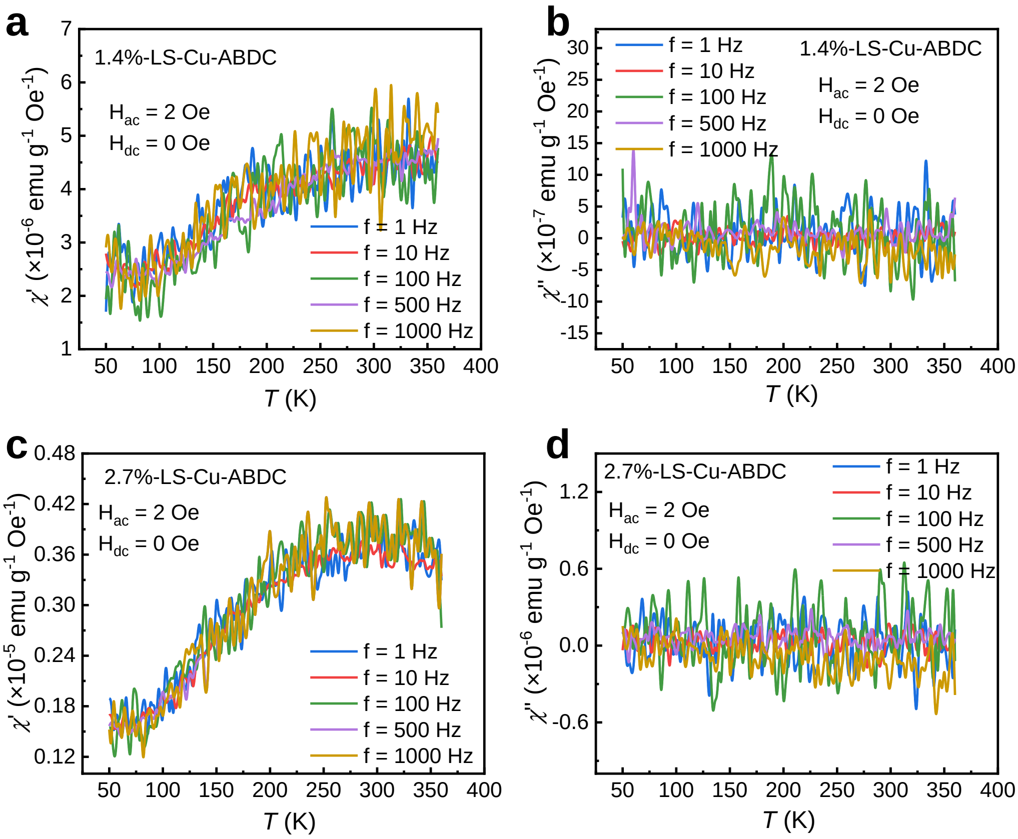

Supplementary Figure 21. AC magnetic susceptibility measurement of 1.4%- and 2.7%-LS-Cu-ABDC MOF. (**a-b**) Temperature variation of the real part of the ac susceptibility measurement (**a**) and imaginary part (**b**) at various frequencies from 1 Hz to 1000 Hz with H_{ac} = 2 Oe and H_{dc} = 0 Oe from 50 to 360 K for 1.4%-LS-Cu-ABDC MOF. (**c-d**) Temperature variation of the real part of the ac susceptibility measurement (**c**) and imaginary part (**d**) at various frequencies from 1 Hz to 1000 Hz with H_{ac} = 2 Oe and H_{dc} = 0 Oe from 50 to 360 K for 2.7%-LS-Cu-ABDC MOF.

In order to further investigate the long-range ferromagnetic order, we preformed the in-phase and out-of-phase AC magnetic susceptibility $\chi'(T)$ and χ'' (T) for 1.4%- and 2.7%-LS-Cu-ABDC MOF as a function of temperature at an AC field of H_{ac} = 2 Oe and H_{dc} = 0 Oe with several frequencies (f = 1, 10, 100, 500, and 1000 Hz) at the temperature range of 50-360 K. However, we did not observe any frequency-dependent peaks in the AC magnetic susceptibility characterizations, which are often attributed to short-range behavior such as spin glass.[30-32] Meanwhile, AC magnetic susceptibility signals related to long-range order were also missing in the measurements, probably due to that the test temperature is well below the transition temperature T_C or the response of our samples to AC magnetic susceptibility is relatively weak because of the weak magnetism.

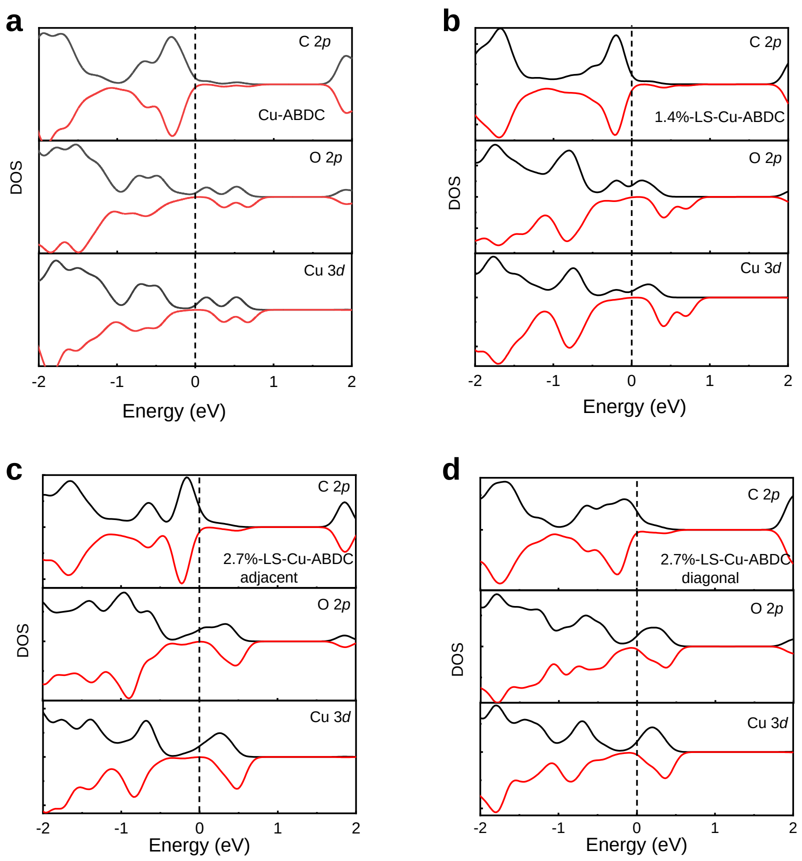

Supplementary Figure 22. Theoretical calculation. The calculated densities of states (DOS) for Cu-ABDC (**a**), 1.4%-LS-Cu-ABDC (**b**) and 2.7%-LS-Cu-ABDC (**c-d**), respectively. DFT calculations reveal that the long-range ferromagnetic interaction between adjacent dimers mainly arises from the hybridization between Cu 3*d*, O 2*p* and C 2*p* orbitals.

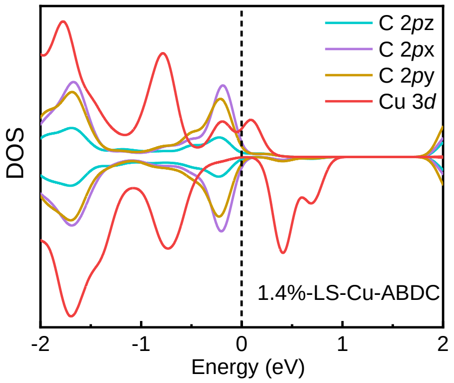

Supplementary Figure 23. The calculated partial densities of states (PDOS) for 1.4%-LS-Cu-ABDC. The PDOS near the Fermi level of 1.4%-LS-Cu-ABDC display increased hybridization of the orbitals from Cu(3*d*) and C(2*pz*), indicating the presence of delocalized π electrons and π symmetry.

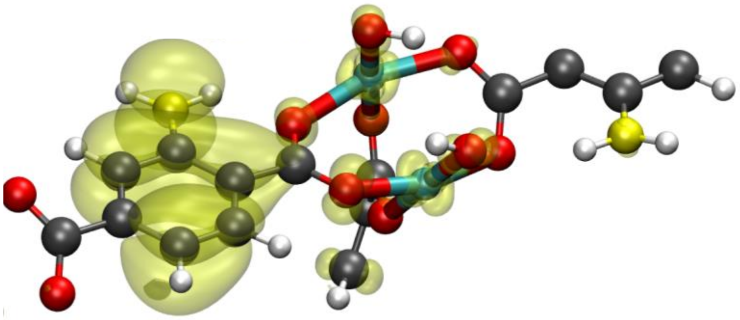

Supplementary Figure 24. Spatial distribution of one of the molecular orbitals with π symmetry for1.4%-LS-Cu-ABDC. It displays clear hybridization between the orbitals from C(2*pz*) and Cu(3*d*), indicating the presence of delocalized π electrons and π symmetry. Yellow iso-surfaces represent the spatial distribution of the C(2*pz*) orbital. Copper, carbon, nitrogen, oxygen and hydrogen atoms are shown in blue, gray, yellow, red and white, respectively, and the axial solvent molecules are omitted for clarity.

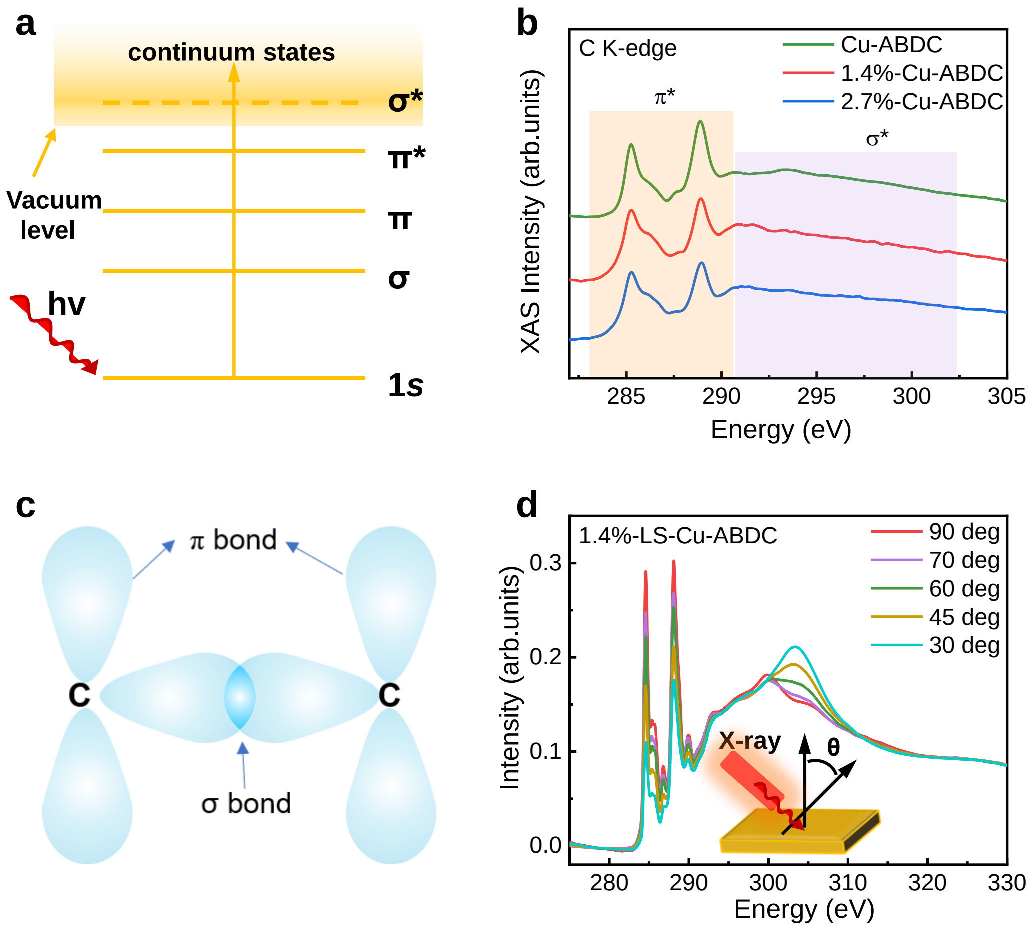

Supplementary Figure 25. Schematic illustration of an X-ray adsorption process of a diatomic molecule (**a**). C K-edge XAS of Cu-ABDC MOFs (**b**). The shadings were used to exhibit different regions. The schematic of π and σ bond (**c**). Angle-dependent XAS spectra of 1.4%-Cu-ABDC MOF at C K-edge (**d**). The measurement geometry is shown as an insert.

The relative intensities and energy of the observed features remain similar between all Cu-ABDC materials while their magnetism and electronic structures are very different. The reason is that the C K-edge XAS represents the transition from C 1*s* to unoccupied molecular orbitals with C 2*p* components (Figure S25a), and the magnetism of Cu-MOF are mainly related to the hybrid between Cu 3*d* orbitals. C K-edge XAS depends directly on the local coordination environment of C atoms, and the cleaving strategy mainly alters the local coordinated environment of the Cu atoms, resulting in slight changes in the overall lattice structure of the MOF. Therefore, the relative intensities and energy of the observed features in C K-edge XAS remain similar between all Cu-ABDC materials. Additionally, the C K-edge XAS spectra (Figure S25b) of Cu-ABDC MOFs can be roughly divided into two regions according to the incident

photon energy due to the random orientation of the samples. Before 293 eV can be assigned to the π^* region (marked as yellow shadings), and in higher energy regions above 293 eV can be attributed to σ^* region (marked as purple shadings).[33-36] Specifically, the photon energy position of π^* in our samples indicates electronic transitions from C 1*s* core level to delocalized π^* energy level in Cu-ABDC MOFs, which suggests the delocalized π electrons are present in our MOFs.

To obtain the angle-dependent C K-edge XAS spectra, the Cu-MOF was carefully dissolved in ethanol and spin-coated on a silicon wafer to fabricate an oriented-aggregated sample. Due to the π-π stacking interaction, the Cu-MOF sheets prefer to be deposited on silicon wafer with the benzene ring roughly perpendicular to the substrate. In other words, the aromatic ring of Cu-MOF is along the normal of the sample. Hence, we can utilize the linear polarized X-ray to district the orbitals with π and σ symmetry (Figure S25c). The region before 293 eV where the intensity increases with the angle of incident light can be assigned to π^* orbitals. The highest intensity was obtained when the angle between the electric field vector **E** of the incident light and the normal of the sample is 90°, indicating that the final states are the orbitals perpendicular to the aromatic ring plane, e.g. the π orbitals. In higher energy σ^* region above 293 eV, the intensity decreases with the angle of incident light. Here, the final state of transition is within the aromatic ring plane, which is with σ symmetry. In other similar systems such as graphene oxide and cobalt phthalocyanine, π and σ orbitals are also assigned at similar energies.[37-39]

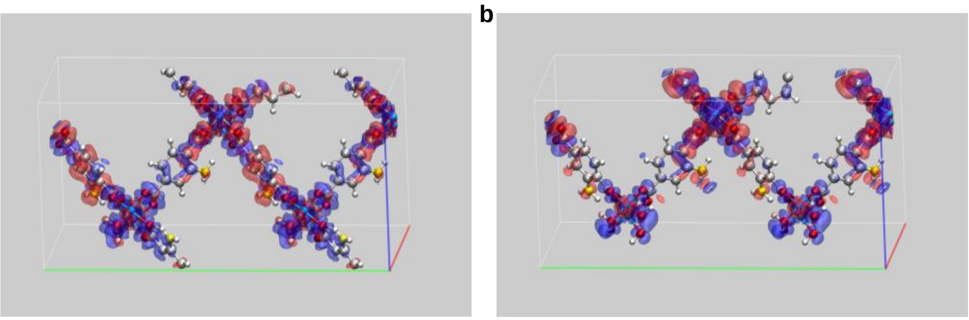

Supplementary Figure 26. Theoretical calculation results on a larger unit-cell for 1.4%-LS-Cu-ABDC (a) and 2.7%-LS-Cu-ABDC with the adjacent ligands (b), respectively. Red and blue iso-surfaces represent positive and negative spin densities, and the value is 0.0002 spins per $bohr^3$, Copper, carbon, nitrogen, oxygen and hydrogen atoms are shown in blue, gray, yellow, red and white, respectively, and the axial solvent molecular are omitted for clarity in all structure models.

To confirm the long-range ferromagnetic order in our samples, theoretical calculation results on a larger unit-cell were performed. The spatial distribution of spin densities on a large unit-cell was shown in Figure S26. We can clearly see that the spin states are present in the benzene ring of the organic linkers, indicating that the delocalized π electrons in the organic linkers provide the bridge for the exchange interaction between Cu dimers. After the substitution of ligands, the spatial distribution of spin densities on the organic linkers is always present, and just gets weaker with the cleavage of the linkers. And the spin densities of the axial solvent molecules and partial atoms are omitted for clarity in all structure models.

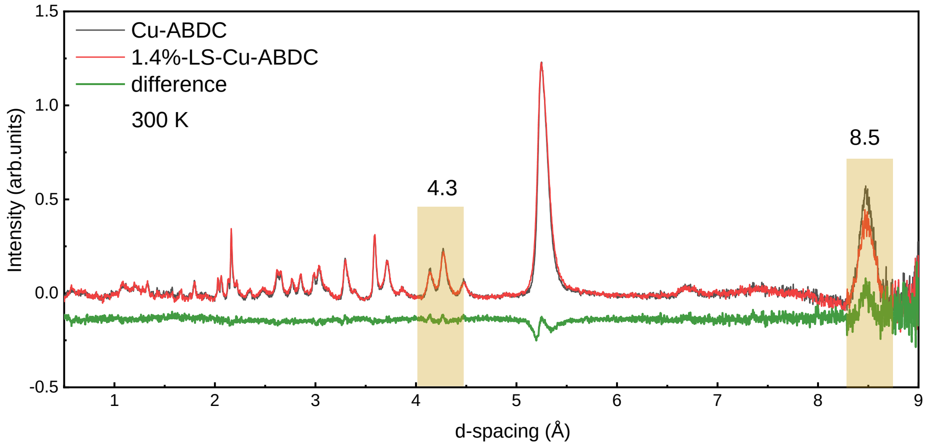

Supplementary Figure 27. Neutron diffraction patterns for Cu-ABDC and 1.4%-LS-Cu-ABDC. The yellow shadings were used to highlight the differences in peaks.

The neutron diffraction measurements were performed at 300 K in the *d*-spacing range of 0.5-9 Å. The intensity of the magnetic contribution to the diffraction peaks will decrease towards higher angles, thus, magnetic scattering peaks are most likely present in lower angles. We have obtained the neutron diffraction patterns of the pristine antiferromagnetic Cu-ABDC and ferromagnetic 1.4%-LS-Cu-ABDC samples. Since the magnetic interactions in our samples are relatively small, the intricate structure and the abundance of hydrogen atoms in our MOFs, prevent it from accurately matching our calculated crystal structure. However, compared to that of the pristine antiferromagnetic Cu-ABDC, the neutron diffraction patterns of 1.4%-LS-Cu-ABDC exhibit a clear decrease at about 8.5 Å with the emergence of the intrinsic ferromagnetism, indicating the contribution of long-range magnetic order.[40-41]

Supplementary Tables

Supplementary Table 1. Elemental analysis by a combination of inductively coupled plasma atomic emission spectrometry (ICP-AES) and C, N and H combustion method for Cu-ABDC and LS-Cu-ABDC MOFs.

Cu-ABDC	N (%)	C (%)	H (%)	Cu (%)	O (%)
Experimental	8.78	41.94	3.95	19.71	25.62
Calculated	8.86	41.77	3.80	20.25	25.32
**1.4%-LS-Cu-ABDC**	**N (%)**	**C (%)**	**H (%)**	**Cu (%)**	**O (%)**
Experimental	8.09	40.48	3.94	19.87	27.62
Calculated	7.90	41.61	3.95	20.65	25.81
**2.7%-LS-Cu-ABDC**	**N (%)**	**C (%)**	**H (%)**	**Cu (%)**	**O (%)**
Experimental	8.76	41.63	4.00	19.91	25.70
Calculated	7.50	43.04	2.85	21.69	24.84

In order to check the validity of the Cu(ABDC)·DMF formula, we performed elemental analysis by a combination of ICP-AES and C, N and H combustion methods, the weight percent is presented in the table. Taking into account of the formula $CuC_{11}N_2O_5H_{12}$, we can obtain the following elemental ratios according to the experiment results: C/H≈11/12.4, C/N≈11/2, C/Cu ≈11/1, C/O≈11/5, which is basically consistent with the theoretical value. Therefore, the element analysis finally validates a chemical formula as Cu(ABDC)·DMF for the layered semiconducting MOF that we have synthesized. We also performed elemental analysis for LS-Cu-ABDC MOFs. Taking into account of the corresponding formula for 1.4%- and 2.7%-LS-Cu-ABDC MOFs according to the results of FT-IR analysis, we can obtain the following elemental ratios according to the experiment results: C/H≈10.75/12.59, C/N≈10.75/1.84, C/O≈10.75/5.1, C/Cu≈10.75/1 for 1.4%-LS-Cu-ABDC, and C/H≈10.58/12.20, C/N≈10.58/1.90, C/O≈10.58/4.9, C/Cu≈10.58/1 for 2.7%-LS-Cu-ABDC, which is basically consistent with the following chemical formula: $Cu(ABDC)_{0.75}\cdot(BA)_{0.25}\cdot DMF\cdot OH_{0.25}$ and $Cu(ABDC)_{0.58}\cdot(BA)_{0.42}\cdot DMF\cdot OH_{0.42}$. Therefore, all the above results indicate that the chemical formula for Cu-ABDC, 1.4%- and 2.7%-LS-Cu-ABDC can be estimated to be Cu(ABDC)·DMF, $Cu(ABDC)_{0.75}\cdot(BA)_{0.25}\cdot DMF\cdot OH_{0.25}$ and $Cu(ABDC)_{0.58}\cdot(BA)_{0.42}\cdot DMF\cdot OH_{0.42}$, respectively. Besides, the atomic ratio of C/Cu is in agreement with the theoretical results, indicating no excess Cu. Therefore, there is also no CuO in our samples.

Supplementary Table 2. The Rietveld refined XRD pattern results. The lattice parameters of the MOF obtained from the refinement are basically in agreement with the reported values, indicating a reliable quality for our samples.

Parameters	Standard	Refined
a **(Å)**	11.41(4)	11.13(4)
b **(Å)**	14.27(8)	14.24(3)
c **(Å)**	7.78(0)	7.85(2)
α **(°)**	90	90
β **(°)**	108.12 (9)	110.99(8)
γ **(°)**	90	90
$\mathbf{R_p}$ **(%)**	-	1.38
$\mathbf{R_{wp}}$ **(%)**	-	1.97

Supplementary Table 3. Cu K-edge EXAFS fitting results for Cu-ADBC MOFs.

Sample	Path	*N*	*R* (Å)	σ^2 ($Å^2$)	ΔE_0	*R*
Cu-ABDC	Cu-O_1	4±0.4	1.95±0.01	0.0057±0.001	-7.1±0.8	0.000058
	Cu-O_2	1.2±0.1	1.98±0.01	0.003±0.001		
	Cu-Cu	1±0.1	2.67±0.01	0.012±0.003		
1.4%-LS-Cu-ABDC	Cu-O_1	3.7±0.3	1.95±0.01	0.005±0.001	-2.0±1.6	0.00015
	Cu-O_2	1.3±0.1	1.98±0.01	0.004±0.001		
	Cu-Cu	1±0.1	2.72±0.01	0.015±0.007		
2.7%-LS-Cu-ABDC	Cu-O_1	3.4±0.3	1.96±0.01	0.0047±0.001	-3.2±10.6	0.00074
	Cu-O_2	1.7±0.2	1.98±0.01	0.004±0.001		
	Cu-Cu	1±0.1	2.75±0.01	0.018±0.003		

N, coordination number; *R*, bonding distance; σ^2, Debye-Waller factor; ΔE_0, inner potential shift; *R* factor reveals the goodness of the fit. The fitted *R* range here is from 1.1 to 2.4 Angstroms.

Supplementary Table 4. DFT calculation results of Cu-ABDC and LS-Cu-ABDC with solvent molecules.

Sample	Structure	E_{FM} (eV)	E_{AFM} (eV)	$\Delta E_{FM\text{-}AFM}$ (eV)	Magetic momet (μ_B)	$d_{Cu\text{-}Cu}$ (Å)
Cu-ABDC	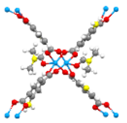	-52720.775	-52720.893	0.118	0.57	2.588
1.4%-LS-Cu-ABDC	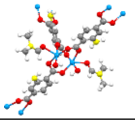	-52516.939	-52516.912	-0.027	0.32	2.693
2.7%-LS-Cu-ABDC (adjacent)	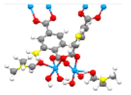	-52312.844	---	---	0.1	2.731
2.7%-LS-Cu-ABDC (diagonal)	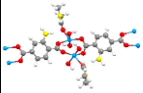	-52312.569	---	---	0.04	2.910

ΔE defined as the difference in energy between the FM and AFM spin configurations: $\Delta E = E_{FM} - E_{AFM}$. The signal "---" represents the absence of antiferromagnetic state. Copper, carbon, nitrogen, oxygen and hydrogen atoms are shown in blue, gray, yellow, red and white, respectively.

Supplementary References

1. Deng, H., Doonan, C.J., Furukawa, H., Ferreira, R. B., Towne, J., Knobler, C. B., Wang, B., Yaghi, O.M. Multiple Functional Groups of Varying Ratios in Metal-Organic Frameworks. *Science* **327**, 846-850 (2010).
2. Braun, M. E., Steffek, C. D., Kim, J., Rasmussen, P. G., Yaghi, O. M. 1,4-Benzenedicarboxylate derivatives as links in the design of paddle-wheel units and metal-organic frameworks. *Chem. Commun.* 2532-2533 (2001).
3. Carson, C. G. et al. Synthesis and Structure Characterization of Copper Terephthalate Metal-Organic Frameworks. *Eur. J.Inorg. Chem.* **2009**, 2338-2343 (2009).
4. Rodenas, T. et al. Metal-organic framework nanosheets in polymer composite materials for gas separation. *Nat. Mater.* **14**, 48-55 (2015).
5. Zhan, G. et al. Fabrication of Ultrathin 2D Cu-BDC Nanosheets and the Derived Integrated MOF Nanocomposites. *Adv. Funct. Mater.* **29**, 1806720 (2019).
6. Souto, M. et al. Breathing-Dependent Redox Activity in a Tetrathiafulvalene-Based Metal-Organic Framework. *J. Am. Chem. Soc.* **140**, 10562-10569 (2018).
7. Pathak, A. et al. Integration of a (-Cu-S-)n plane in a metal-organic framework affords high electrical conductivity. *Nat. Commun.* **10**, 1721 (2019).
8. Zhang, W. et al. Dynamic Restructuring of Coordinatively Unsaturated Copper Paddle Wheel Clusters to Boost Electrochemical CO_2 Reduction to Hydrocarbons. *Angew. Chem. Int. Ed. Engl.* **61**, e202112116 (2022).
9. Zhao, M. et al. Core-shell palladium nanoparticle@metal-organic frameworks as multifunctional catalysts for cascade reactions. *J. Am. Chem. Soc.* **136**, 1738-1741 (2014).
10. Zhang, Y. W., Li, Z., Zhao, Q., Zhou, Y. L., Liu, H. W., Zhang, X. X. A facilely synthesized amino-functionalized metal-organic framework for highly specific and efficient enrichment of glycopeptides. *Chem. Commun.* **50**, 11504-11506 (2014).
11. Dhumal, N. R., Singh, M. P., Anderson, J. A., Kiefer, J., Kim, H. J. Molecular Interactions of a Cu-Based Metal-Organic Framework with a Confined Imidazolium-Based Ionic Liquid: A Combined Density Functional Theory and Experimental Vibrational Spectroscopy Study. *J. Phys. Chem. C* **120**, 3295-3304 (2016).
12. Deng, X. et al. Metal-Organic Framework Coating Enhances the Performance of Cu_2O in Photoelectrochemical CO_2 Reduction. *J. Am. Chem. Soc.* **141**, 10924-10929 (2019).
13. Prestipino, C., Regli, L., Vitillo, J. G., Bonino, F., Damin, A., Lamberti, C., Zecchina, A., Solari, P. L., Kongshaug, K. O., Bordiga, S. Local Structure of Framework Cu(II) in HKUST-1 Metallorganic Framework: Spectroscopic Characterization upon Activation and Interaction with Adsorbates. *Chem. Mater.* **18**, 1337-1346 (2006).
14. Bordiga, S. et al. Electronic and vibrational properties of a MOF-5 metal-organic framework: ZnO quantum dot behaviour. *Chem. Commun.* 2300-2301

(2004).

15. Suntivich, J. et al. Estimating Hybridization of Transition Metal and Oxygen States in Perovskites from O K-edge X-ray Absorption Spectroscopy. *J. Phys. Chem. C* **118**, 1856-1863 (2014).
16. de Groot, F. M., Grioni, M., Fuggle, J. C., Ghijsen, J., Sawatzky, G. A., Petersen, H. Oxygen 1*s* x-ray-absorption edges of transition-metal oxides. *Phys. Rev. B* **40**, 5715-5723 (1989).
17. Chen, Y. et al. Electronic origin of hydrogen storage in MOF-covered palladium nanocubes investigated by synchrotron X-rays. *Commun. Chem.* **1**, 61 (2018).
18. Ziebel, M. E., Darago, L. E., Long, J. R. Control of Electronic Structure and Conductivity in Two-Dimensional Metal-Semiquinoid Frameworks of Titanium, Vanadium, and Chromium. *J. Am. Chem. Soc.* **140**, 3040-3051 (2018).
19. Xiao, J. D., Han, L., Luo, J., Yu, S.H., Jiang, H. L. Integration of Plasmonic Effects and Schottky Junctions into Metal-Organic Framework Composites: Steering Charge Flow for Enhanced Visible-Light Photocatalysis. *Angew. Chem. Int. Ed. Engl.* **57**, 1103-1107 (2018).
20. Darago, L. E., Aubrey, M. L., Yu, C. J., Gonzalez, M. I., Long, J. R. Electronic Conductivity, Ferrimagnetic Ordering, and Reductive Insertion Mediated by Organic Mixed-Valence in a Ferric Semiquinoid Metal-Organic Framework. *J. Am. Chem. Soc.* **137**, 15703-15711 (2015).
21. Pham, H. et al. Imparting Functionality and Enhanced Surface Area to a 2D Electrically Conductive MOF via Macrocyclic Linker. *J. Am. Chem. Soc.* **144**, 10615-10621 (2022).
22. Dong, R. et al. A coronene-based semiconducting two-dimensional metal-organic framework with ferromagnetic behavior. *Nat. Commun.* **9**, 2637 (2018).
23. Goncalves, R. H., Lima, B. H., Leite, E.R. Magnetite colloidal nanocrystals: a facile pathway to prepare mesoporous hematite thin films for photoelectrochemical water splitting. *J. Am. Chem. Soc.* **133**, 6012-6019 (2011).
24. Luo, W., Wang, J., Zhao, X., Zhao, Z., Li, Z., Zou, Z. Formation energy and photoelectrochemical properties of $BiVO_4$ after doping at Bi^{3+} or V^{5+} sites with higher valence metal ions. *Phys. Chem. Chem. Phys.* **15**, 1006-1013 (2013).
25. Liao, L. et al. Efficient solar water-splitting using a nanocrystalline CoO photocatalyst. *Nat. Nanotech.* **9**, 69-73 (2014).
26. Castaldelli, E. et al. Electrical semiconduction modulated by light in a cobalt and naphthalene diimide metal-organic framework. *Nat. Commun.* **8**, 2139 (2017).
27. Liu, X. et al. Photoconductivity in Metal-Organic Framework (MOF) Thin Films. *Angew. Chem. Int. Ed. Engl.* **58**, 9590-9595 (2019).
28. Arora, H. et al. Demonstration of a Broadband Photodetector Based on a Two-Dimensional Metal-Organic Framework. *Adv. Mater.* **32**, e1907063 (2020).
29. Liu, C. K. et al. 2D Metal-Organic Framework $Cu_3(HHTT)_2$ Films for Broadband Photodetectors from Ultraviolet to Mid-Infrared. *Adv. Mater.* **34**, e2204140 (2022).
30. Singh, M. K., Prellier, W., Singhm, M. P., Katiyar, R. S., Scott, J. F. Spin-glass

transition in single-crystalBiFeO_3. *Phys. Rev. B* **77**, (2008).

31. Goremychkin, E. A, Osborn, R., Rainford, B. D., Macaluso, R.T., Adroja, D.T., Koza, M. Spin-glass order induced by dynamic frustration. *Nat. Phys.* **4**, 766-770 (2008).
32. Mustonen, O. et al. Spin-liquid-like state in a spin-1/2 square-lattice antiferromagnet perovskite induced by d(10)-d(0) cation mixing. *Nat. Commun.* **9**, 1085 (2018).
33. Wang, D., Yang, J., Li, X., Geng, D., Li, R., Cai, M., Sham, T.-K., Sun, X. Layer by layer assembly of sandwiched graphene/SnO_2 nanorod/carbon nanostructures with ultrahigh lithium ion storage properties. *Energy Environ. Sci.* **6**, 2900 (2013).
34. Liang, Y. et al. Covalent hybrid of spinel manganese-cobalt oxide and graphene as advanced oxygen reduction electrocatalysts. *J. Am. Chem. Soc.* **134**, 3517-3523 (2012).
35. Liu, L. et al. Spectroscopic Identification of Active Sites of Oxygen-Doped Carbon for Selective Oxygen Reduction to Hydrogen Peroxide. *Angew. Chem. Int. Ed.*, **62**, e202303525 (2023).
36. Han, G. F. et al. Building and identifying highly active oxygenated groups in carbon materials for oxygen reduction to H_2O_2. *Nat. Commun.* **11**, 2209 (2020).
37. Basagni, A. et al. Tunable Band Alignment with Unperturbed Carrier Mobility of On-Surface Synthesized Organic Semiconducting Wires. *ACS Nano* **10**, 2644-51 (2016).
38. O'Donnell, K. M. et al. Orientation and stability of a bi-functional aromatic organic molecular adsorbate on silicon. *Phys. Chem. Chem. Phys.* **18**, 27290-27299 (2016).
39. Su, G. M., Patel, S. N., Pemmaraju, C. D., Prendergast, D., Chabinyc, M. L. First-Principles Predictions of Near-Edge X-ray Absorption Fine Structure Spectra of Semiconducting Polymers. *J. Phys. Chem. C* **121**, 9142-9152 (2017).
40. Kozlenko, D. P. et al. Spin-induced negative thermal expansion and spin-phonon coupling in van der Waals material $CrBr_3$. *npj Quantum Mater.* **6** (2021).
41. Hirai, S., dos Santos, A. M., Shapiro, M. C., Molaison, J. J., Pradhan, N., Guthrie, M., Tulk, C. A., Fisher, I. R.; Mao, W. L. Giant atomic displacement at a magnetic phase transition in metastable Mn_3O_4. *Phys. Rev. B* **87** (2013).
